# Clínica neurológica fluctuante: ¿Llamo al neurólogo o al hematólogo?

**DOI:** 10.1515/almed-2020-0030

**Published:** 2020-08-17

**Authors:** Rita Losa-Rodríguez, Carmen Pérez Martínez, Gabriel Rodríguez Pérez, Ignacio de la Fuente Graciani, Lara M. Gómez García

**Affiliations:** Servicio de Análisis Clínicos, Hospital Clínico Universitario de Valladolid, Valladolid, España; Servicio de Hematología y Hemoterapia, Hospital Clínico Universitario de Valladolid, Valladolid, España

**Keywords:** esquistocitos, microangiopatía trombótica, trombocitopenia

## Abstract

**Objetivos:**

Resaltar el papel del laboratorio clínico y la pronta comunicación con el servicio de Hematología en el diagnóstico y la rapidez en la instauración del tratamiento de una patología hematológica tan urgente como la Púrpura Trombótica Trombocitopénica (PTT).

**Caso clínico:**

Varón de edad avanzada derivado a Urgencias hospitalarias por su médico de Atención Primaria por trastorno de emisión del lenguaje, asimetría facial y disminución de fuerza en extremidad superior, por lo que se activa el código ictus. Sin embargo, los hallazgos de laboratorio (anemia y trombocitopenia, creatinina, bilirrubina total y LDH elevadas, test de Coombs directo negativo) y presencia de esquistocitos en el frotis de sangre periférica, acaban derivando en un diagnóstico completamente diferente: microangiopatía trombótica tipo PTT.

**Conclusiones:**

La primera orientación diagnóstica de ictus hemisférico izquierdo fue rechazada ante los signos de anemia hemolítica no autoinmune, trombocitopenia sin causa aparente y presencia de esquistocitos. No debemos olvidar que esta patología puede cursar con afectación multiorgánica y características clínicas muy variables. Aunque su diagnóstico definitivo se basa en la determinación de actividad de ADAMTS-13, debido a la elevada mortalidad es necesario instaurar tratamiento de forma inmediata tras su sospecha.

## Introducción

La púrpura trombótica trombocitopénica (PTT) es una entidad hematológica aguda y potencialmente mortal caracterizada por anemia hemolítica microangiopática y trombocitopenia. La presentación clínica característica deriva de la propia fisiopatología: anemia hemolítica no autoinmune, trombocitopenia severa, manifestaciones neurológicas fluctuantes, afectación renal y fiebre; aunque estas dos últimas son menos frecuentes [1], [2].

Puede darse de manera congénita o adquirida, apareciendo como consecuencia de un déficit o disfunción de la proteína *disintegrin and metalloproteinase with a thrombospondin type 1 motif, member 13* (ADAMTS13). Esta proteasa es responsable de la escisión de los multímeros de alto peso molecular del Factor de von Willebrand (FvW). Los multímeros de FvW no degradados tienen una gran afinidad por las plaquetas, formando trombos que terminan obstruyendo los vasos de la microcirculación y originando daño endotelial en forma de isquemia, afectando especialmente a la microcirculación cerebral y renal. Esto origina trombocitopenia severa por consumo plaquetario, diátesis hemorrágica, anemia microangiopática y daño orgánico [1], [3].

La sospecha diagnóstica de una microangiopatía trombótica (MAT) se debe plantear ante la presencia de anemia hemolítica por fragmentación de hematíes (esquistocitos) y trombocitopenia sin causa aparente, junto con la posible presencia de signos y síntomas de daño orgánico. Ante el diagnóstico de una MAT, se debe considerar a la PTT como primera entidad posible e iniciar tratamiento de forma inmediata con recambios plasmáticos y corticoides, hasta disponer de los resultados de ADAMTS-13 que confirmen el diagnóstico de PTT [4].

## Caso clínico

Varón de 86 años derivado al servicio de Urgencias hospitalarias por su médico de Atención Primaria, por trastorno de emisión del lenguaje y asimetría facial de varios minutos, así como disminución de fuerza en extremidad superior derecha, sin remisión total de la clínica. Posteriormente en Urgencias reaparece la misma sintomatología por lo que se activa el código ictus, siendo valorado por el equipo de Neurología de guardia.

En la exploración general destaca la presencia de petequias en extremidades y tórax, ictericia conjuntival y afectación neurológica leve (NIHSS: 3 puntos) según escala del ictus del Instituto Nacional de la Salud.

En la analítica destaca bicitopenia marcada (hemoglobina 81 g/L [120–180 g/L] y plaquetas 16 × 10^3^/μL [150–400 × 10^3^/μL]) que se confirma en frotis de sangre periférica (FPS), sin poder visualizarse esquistocitos. Bioquímica alterada (creatinina 1,54 mg/dL [0,7–1,2 mg/dL], bilirrubina total 2,53 mg/dL [0,10–1,20 mg/dL] a expensas de bilirrubina indirecta, LDH 1.110 UI/L [135–250 UI/L]) y test de Coombs directo negativo. No alteración iónica ni de la función hepática.

En el TAC cerebral no se observan signos precoces de isquemia y hemorragia. En el angioTAC no se observa oclusión extra ni intracraneal y los mapas de perfusión cerebral no presentan alteraciones. Sin embargo, el paciente continúa con clínica compatible con ictus hemisférico izquierdo presentando fluctuaciones. Ante los hallazgos analíticos, se pospone el inicio de antiagregación y se mantiene la vigilancia en la unidad de ictus. En este contexto clínico, se solicita valoración por parte del servicio de Hematología que plantea como primera posibilidad un cuadro de MAT.

**Figura 1:**
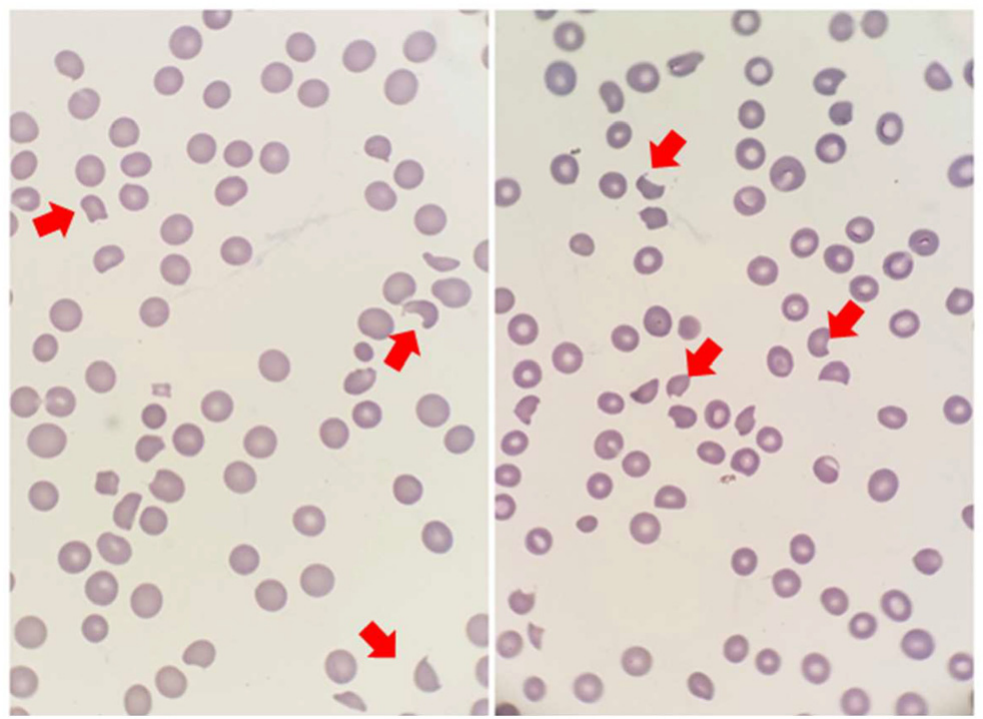
Frotis de sangre periférica al diagnóstico: anisopoiquilocitosis con presencia de esquistocitos y trombocitopenia confirmada.Las flechas rojas indican la morfología típica de esquistocitos. Tinción May-Grunwald-Giemsa. Microscopía óptica, ×1.000 aumentos.

Se solicita un nuevo control analítico, destacando la severidad de la anemia (hemoglobina 74 g/L) y la persistencia de trombocitopenia severa (13000/μL; sin modificaciones en citrato), así como hemoglobinuria intensa en tira reactiva de orina. En esta ocasión, el estudio del FSP revela la presencia de anisopoiquilocitosis y esquistocitos (Figura 1).

Con todos estos datos clínicos y analíticos, el cuadro clínico parece compatible con MAT tipo PTT, habiéndose descartado previamente otras causas secundarias de MAT.

Se decide inicio inmediato de corticoterapia intensiva a dosis de 2 mg/kg/día y se programan recambios plasmáticos terapéuticos (RPT) diarios, previa extracción de muestras para determinación de ADAMTS13. El valor de actividad de ADAMTS13 resultó de un 0% (actividad de ADAMTS13 < 5% es indicativa de PTT) con presencia de inhibidor de ADAMTS13 [5].

Se realizan RPT diarios, utilizándose como solución de reposición plasma fresco congelado (PFC), hasta el 6° día, objetivándose un aumento progresivo en la cifra plaquetaria hasta alcanzar valores de >150 × 10^3^/μL en 2 días consecutivos, modificándose posteriormente el régimen de RPT cada 48 horas, sin presentar descenso plaquetario.

El paciente presenta buena tolerancia a RPT, presentando únicamente parestesias periorales y en punta de dedos de manos y pies, consecuencia de la hipocalcemia de consumo por el citrato utilizado en RPT, precisando de monitorización analítica estrecha y remitiendo con suplementos de gluconato cálcico.

**Figura 2:**
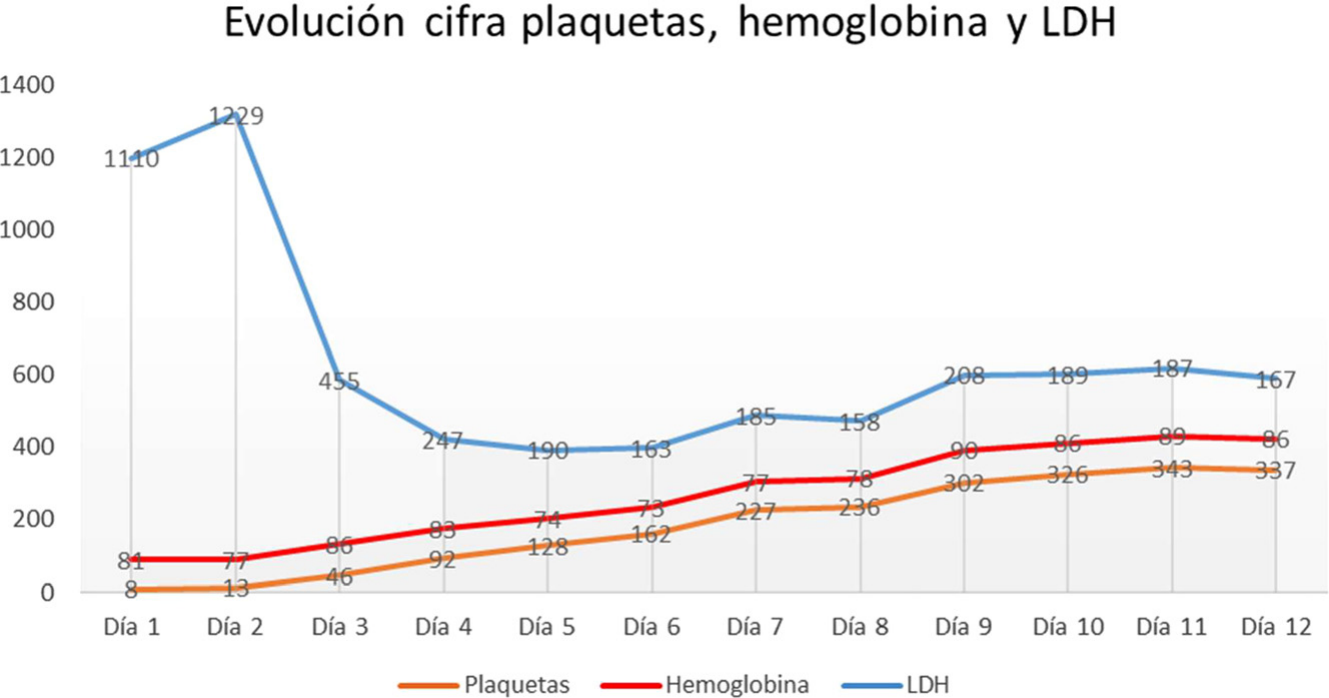
Evolución cifra de plaquetas “×10^3^/μL”, hemoglobina “g/L” y LDH “UI/L” desde el diagnóstico hasta la finalización de los RPT.

La evolución clínica resulta favorable, con normalización de cifras plaquetarias y anemia (Figura 2), desaparición de datos de hemólisis y permaneciendo el paciente asintomático tras recibir 8 sesiones de RPT. Finalmente, el paciente es dado de alta para control en consulta con mantenimiento de corticoterapia a dosis de 1 mg/kg/día, con posterior pauta de descenso progresivo de corticoides hasta su suspensión.

## Discusión

La incidencia de PTT en EEUU es de 4–6 pacientes por millón de habitantes y año. En España no existen todavía datos exactos, aunque se está llevando a cabo un registro nacional por el Grupo Español de PTT. El curso natural de PTT sin tratamiento lleva a la muerte hasta un 85–90% de los pacientes, y se debe principalmente a los eventos isquémicos. Además, una parte considerable de los supervivientes ve afectada su calidad de vida, con síndromes depresivos y déficits neurocognitivos [6], [7], [8].

En más del 90% de los casos es idiopática, aunque en una minoría de pacientes se ha asociado al tratamiento farmacológico (ticlopidina, clopidogrel, tacrolimus), enfermedades autoinmunes, o al embarazo. En menos del 5% es congénita (Síndrome de Upshaw-Schulman), debido a mutaciones en el gen codificante de ADAMTS13 siendo más grave en la infancia y adolescencia [9].

El diagnóstico inicial de la PTT es clínico, apoyado en los datos de laboratorio sugestivos de dicha patología. La forma de presentación más frecuente es la diátesis hemorrágica (66%) seguida de las alteraciones neurológicas (66%) y fiebre (24%). Se debe tener en cuenta la cifra plaquetaria, sospechando la entidad en casos de trombocitopenia severa (<50 × 10^3^/μL) con la presencia de esquistocitos en FSP; además de valores indicativos de anemia hemolítica no autoinmune: hemoglobina disminuida, reticulocitos elevados, LDH elevada, hiperbilirrubinemia con elevación de la fracción indirecta, haptoglobina disminuida, Coombs directo negativo y, acompañado o no de incremento moderado de creatinina y hemoglobinuria [10], [11], [12].

El examen del FSP es especialmente relevante en esta entidad, puesto que la presencia de esquistocitos es el dato analítico necesario para el diagnóstico diferencial. Los esquistocitos son producidos por la fragmentación mecánica de los hematíes en las zonas de flujo turbulento próximas a los trombos microvasculares de la PTT. Pueden verse en otras situaciones que dan lugar a condiciones reológicas similares, como prótesis valvulares disfuncionantes, implantes intravasculares, metástasis intravasculares de algunos adenocarcinomas, microangiopatía asociada al trasplante de progenitores hematopoyéticos, enfermedades autoinmunes, infecciones, asociadas a fármacos…; por lo que su presencia es necesaria pero no suficiente para confirmar el diagnóstico. Sin embargo, la presencia de más de 1 esquistocito cada 100 hematíes es altamente sugestiva de MAT [13], [14].

La determinación de la actividad de ADAMTS13 confirma el diagnóstico de PTT cuando existe un déficit absoluto (actividad <5%) tanto en la forma congénita como adquirida de la enfermedad, permitiendo distinguir entre ambos subtipos la presencia de autoanticuerpos anti-ADAMTS13, que son negativos en la forma congénita. Sin embargo, no es todavía una prueba rutinaria en todos los laboratorios, por lo que se envía a un laboratorio de referencia y el resultado puede no estar disponible hasta 24–48 horas después. En línea con lo anterior podemos apoyarnos en una herramienta de diagnóstico, denominada *PLASMIC score*. Este algoritmo utiliza un sistema de puntuación basado en variables predictivas de deficiencia severa de ADAMTS13 (<10% de actividad), y que por tanto estratifica el riesgo de PTT adquirida. Estas variables incluyen la cifra plaquetaria, datos de hemólisis, volumen corpuscular medio, INR, creatinina sérica, historia de cáncer y el trasplante hematopoyético o de órgano sólido [15], [16].

En cuanto a los factores de mal pronóstico, destaca el aumento de LDH (>10 veces límite superior de la normalidad), edad avanzada y aumento de troponina T ultrasensible al diagnóstico (>0,25 ng/dL) y mediante su monitorización, para evaluar el compromiso miocárdico en el paciente con diagnóstico de PTT, aún en un estadío asintomático [17].

La PTT es una urgencia hematológica y debe iniciarse el tratamiento lo antes posible. Ante una anemia hemolítica microangiopática y trombocitopenia, en ausencia de otras causas, debe sospecharse una PTT e iniciarse tratamiento inmediatamente. El tratamiento de elección son los RPT con reposición de plasma, que permiten eliminar los anticuerpos anti-ADAMTS13 del paciente, así como aportar la enzima deficitaria. Debe acompañarse de corticoterapia, puesto que la inmunosupresión actúa inhibiendo los anticuerpos anti-ADAMTS13. Diversos estudios han demostrado su eficacia reduciendo la morbilidad, al disminuir el número de RPT necesarios [4], [18].

Otras opciones terapéuticas son la administración de fármacos biológicos. Rituximab (anti-CD20, utilizado en síndromes linfoproliferativos y enfermedades autoinmunes) en combinación con RPT indicado en etapas precoces de la enfermedad, en pacientes con alto riesgo de mortalidad (afectación neurológica o cardíaca) y relacionado con una menor tasa de recidivas y una más pronta respuesta, así como en casos refractarios y asintomáticos. Y el más novedoso Caplacizumab (inhibe la interacción entre FvW-plaquetas) recientemente aprobado por la AEMPS, planteándose en combinación con RPT e inmunosupresión y asociándose con una normalización más rápida del recuento plaquetario, una menor incidencia de mortalidad y recurrencia de PTT [10], [19], [20].

En los últimos años el avance en el conocimiento fisiopatológico de la enfermedad nos ha permitido profundizar tanto en el diagnóstico precoz como en el abordaje terapéutico inmediato. Gracias a ello, somos capaces de ofrecer un mejor pronóstico de la patología con una menor morbimortalidad.
